# Improvement of Alveolar Bone in a Child with Severe Congenital Neutropenia: Long-Term Clinical Outcomes

**DOI:** 10.3390/dj14060355

**Published:** 2026-06-09

**Authors:** Tatsuya Akitomo, Satoru Kusaka, Jimei Zhao, Ryota Nomura

**Affiliations:** 1 Department of Pediatric Dentistry, Graduate School of Biomedical and Health Sciences, Hiroshima University, Hiroshima 734-8553, Japan; rnomura@hiroshima-u.ac.jp; 2 Department of Pediatric Dentistry, Hiroshima University Hospital, Hiroshima 734-8551, Japan; kusaka-s@cc.osaka-dent.ac.jp (S.K.); choukeimi@hiroshima-u.ac.jp (J.Z.); 3 Department of Pediatric Dentistry, School of Dentistry, Osaka Dental University, Osaka 573-1121, Japan

**Keywords:** alveolar bone loss, child, neutropenia

## Abstract

**Background/Objectives**: Although gingivitis is the most common oral disease in children, periodontitis accompanied by alveolar bone resorption may develop in patients with severe congenital neutropenia. However, no reports to date have focused on changes in the alveolar bone of these patients during long-term follow-up. **Case Summary**: A girl aged 8 years and 5 months who developed leukemia due to severe neutropenia was admitted to the hospital and referred to the pediatric dentistry department for oral care. Panoramic radiographs at the first visit revealed significant alveolar bone resorption and mobility in the remaining deciduous teeth. We provided oral care, and the patient later underwent a hematopoietic stem cell transplant. No oral mucositis was observed. Measurement of alveolar bone thickness in the anterior and posterior regions revealed that the ratio increased as the patient’s systemic condition improved, showing a relative increase in alveolar bone thickness in the posterior region. **Conclusions**: Although this report is descriptive and observational, the patient’s alveolar bone loss with severe congenital neutropenia improved as the patient’s systemic condition improved. In addition, improvement of alveolar bone loss was observed along with systemic recovery and tooth eruption.

## 1. Introduction

Periodontal disease, along with dental caries, is one of the most common oral diseases, and is a prevalent chronic inflammatory condition affecting over a billion people worldwide [1,2]. Periodontal disease is classified into gingivitis and periodontitis. Gingivitis is a reversible disease caused by bacterial biofilms that accumulate on teeth adjacent to the gingiva, while periodontitis results in the loss of connective tissue and bone support, and is a major cause of tooth loss. Pathogenic microorganisms in biofilms, as well as genetic and environmental factors, are involved. Additionally, genetic, dermatological, hematological, granulomatous, immunosuppressive, and neoplastic disorders can also have periodontal manifestations [3]. Although gingivitis is highly prevalent in children and adolescents, severe periodontitis in children is observed as a manifestation of hematologic or genetic disorders such as acquired neutropenia and leukemias, familial and cyclic neutropenia, or Papillon-Lefèvre syndrome [4,5].

Severe congenital neutropenia is one of a group of inherited disorders of hematopoiesis characterized by impaired differentiation of neutrophilic granulocytes and defined by an absolute neutrophil count of <0.5 × 10^9^/L [6]. The estimated prevalence is approximately one to two cases per million with equal distribution by sex [4]. These patients often present with infections such as otitis, skin infections, pneumonia, deep abscesses and septicemia, and have an increased risk of developing leukemia [6]. Periodontitis is another common manifestation, with symptoms ranging from marginal gingivitis to rapidly progressing periodontitis, which involves progressive alveolar bone resorption around both primary and permanent teeth [7]. Although periodontal disease is a lifelong challenge for these patients, there is a lack of adequate information in the dental literature regarding its management because of the rarity of this disease [8].

The periodontal condition of patients with periodontitis associated with systemic diseases can be improved by stabilizing the underlying systemic condition and providing proper oral care [9]. Although many case studies have described periodontal disease or alveolar bone loss in patients with severe congenital neutropenia, few studies have reported changes in the alveolar bone [10,11]. We encountered a patient with severe congenital neutropenia who had developed into leukemia and provided long-term oral management. This report describes the oral management of the patient and demonstrates changes in the alveolar bone through time-dependent radiographic examinations.

## 2. Case Presentation

A girl aged 8 years and 5 months had been under observation for severe congenital neutropenia at another hospital. She had presented with anemia, and detailed examination had confirmed a diagnosis of acute myeloid leukemia. She was subsequently admitted to our hospital for hematopoietic stem cell transplantation and was referred to the pediatric dentistry department for perioperative oral management.

**Figure 1:**
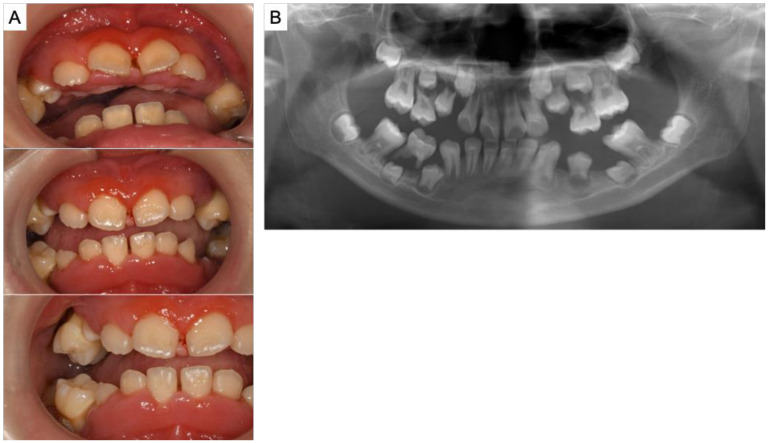
Initial examination at the age of 8 years and 5 months. (**A**) Intraoral photographs. (**B**) Panoramic radiograph.

Nineteen teeth were present in the oral cavity, including three primary second molars (mandibular left primary second molar missing) (Figure 1A). The roots of the primary teeth were exposed, and the teeth had significant mobility, consistent with Miller’s classification as mobility grade II. Additionally, the gingiva around the permanent incisors was red and swollen. Panoramic radiography revealed alveolar bone resorption and radiolucencies around the primary molars (Figure 1B). Although extraction of the primary teeth was indicated, the patient’s systemic condition was very poor (RBC: 2.49 × 10^6^/μL, WBC: 2.65 × 10^3^/μL, PLT: 159 × 10^3^/μL, CRP: 12.42 mg/dL), and the risk of post-extraction infection was high. We consulted with a pediatrician and decided to prioritize improving the patient’s systemic condition while continuing oral care. During the 6-month hospital stay, oral care was continued in the hospital room once a week. In brief, professional tooth cleaning was performed by a dentist or dental hygienist to remove dental plaque, and we instructed parents on how to brush her teeth. No mucositis was observed, and there were no changes in the primary teeth. Because the patient’s systemic condition improved, the hematopoietic stem cell transplantation was not performed, and the patient was discharged.

The patient later received treatment at another hospital, but her systemic condition worsened. She was readmitted to our hospital for tracheostomy for upper airway obstruction at the age of 9 years and 1 month. The maxillary left primary second molar had exfoliated at our reexamination. Radiation therapy was scheduled to begin in 3 weeks, followed by a hematopoietic stem cell transplant in 1 month. We consulted with a pediatrician and decided to extract the primary teeth to eliminate any source of infection. The result of the blood test at that point was described below: RBC: 2.90 × 10^6^/μL, WBC: 4.03 × 10^3^/μL, PLT: 75 × 10^3^/μL, CRP: 3.16 mg/dL. The maxillary and mandibular right primary molars were extracted in the patient’s room using local anesthesia. Postoperative hemostasis and wound healing were uneventful. At the age of 9 years and 2 months, Total Body Irradiation was administered at 3.6 Gy/2 Fr, and bone marrow transplantation was performed as planned following a conditioning regimen with Fludarabine, Etoposide, Anti-thymocyte globulin, and L-PAM. Although we continued oral care once or twice a week, no severe mucositis occurred in the oral cavity. After receiving seven donor lymphocyte infusions, the patient’s condition stabilized and she was discharged at the age of 9 years and 10 months.

**Figure 2:**
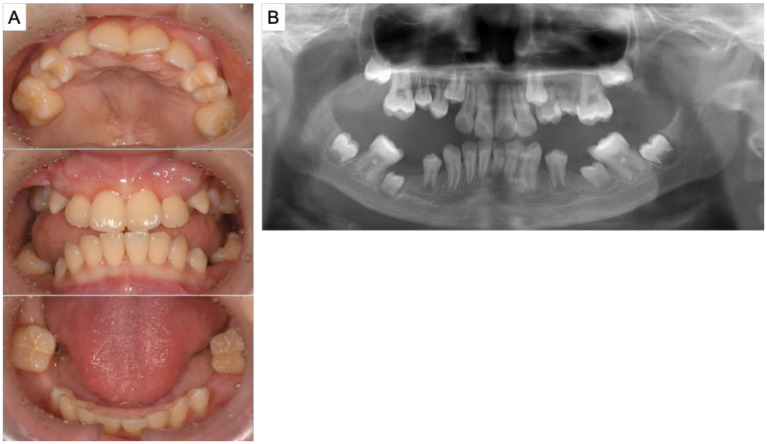
Images at the age of 10 years, showing improvement in the inflammation of the periodontal tissues. (**A**) Intraoral photographs. (**B**) Panoramic radiograph.

During the patient’s hospitalization, the maxillary right second premolar erupted at the age of 9 years and 2 months, and the left one erupted 3 months later. The intraoral photographs taken during the initial outpatient visit at the age of 10 years are shown in Figure 2A. The main blood test results were within the normal range (RBC: 4.25 × 10^6^/μL, WBC: 4.28 × 10^3^/μL, PLT: 218 × 10^3^/μL, CRP: 0.10 mg/dL). No pathological findings, such as dental caries or gingivitis, were observed. Panoramic radiographic examination showed improvement in the width of the alveolar bone (Figure 2B). However, only the anterior teeth were in contact, posing a risk of occlusal trauma. Because the patient lived far away at that time and visited our hospital only once a year, we consulted with an orthodontist at a nearby university hospital. The patient received an orthodontic examination and opted for observation rather than orthodontic treatment.

**Figure 3:**
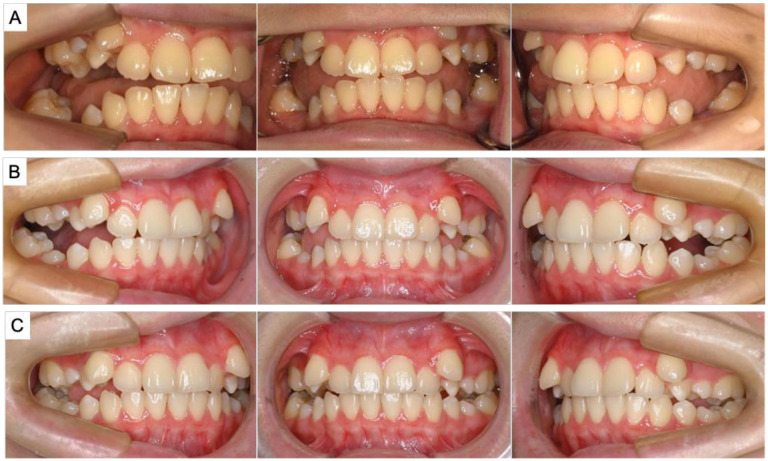
Intraoral photographs. (**A**) 10 years and 10 months, (**B**) 12 years and 8 months, (**C**) 14 years and 8 months.

**Figure 4:**
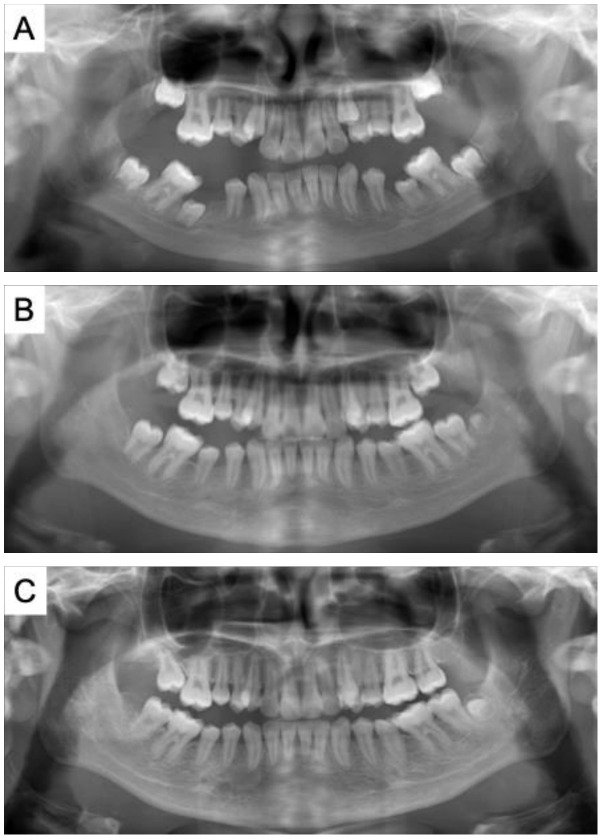
Longitudinal panoramic radiographs showing the increased thickness of the alveolar bone in the molar region. (**A**) 10 years and 10 months, (**B**) 12 years and 8 months, (**C**) 14 years and 8 months.

We continued to monitor the patient’s condition every few years in conjunction with pediatric hospital visits. As tooth eruption progressed, occlusal contact was restored in the molar region (Figure 3A–C). Radiographs taken over time also showed that the thickness of the alveolar bone gradually increased in conjunction with the eruption of the permanent teeth (Figure 4A–C).

**Figure 5:**
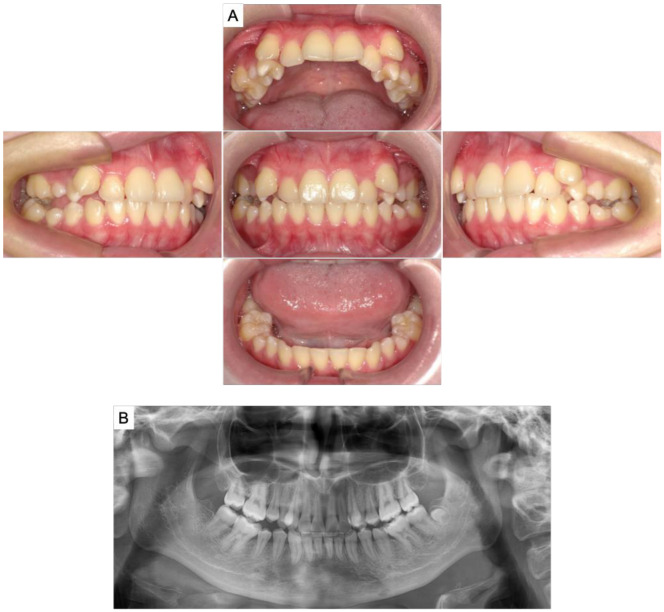
Images at the age of 16 years and 4 months, and no abnormalities were observed in the periodontal tissues. (**A**) Intraoral photographs. (**B**) Panoramic radiograph.

At the age of 16 years and 4 months, 28 permanent teeth had erupted in the oral cavity, and no pathological findings were observed except partial hypoplasia of the first premolars (Figure 5A). Additionally, there were no abnormalities in the periodontal tissue, and the periodontal probing depths were within the normal range. Panoramic radiographs also showed no abnormalities in the alveolar bone (Figure 5B).

**Figure 6:**
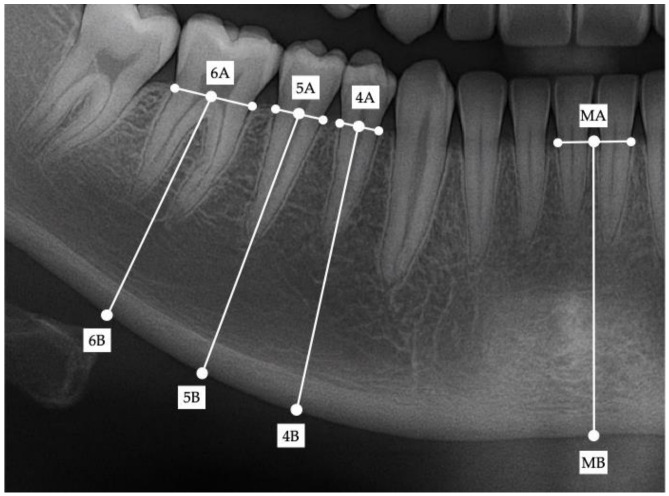
Landmark and reference lines used in the study (This panoramic image was generated by generative artificial intelligence). The numbers indicate the tooth types (4: first premolar, 5: second premolar, 6: first molar), whereas the definitions of the other landmark, such as A and B, are described in the main text.

We evaluated changes in the alveolar bone over time using secular panoramic photographs. The measurement items used in this study are shown in Figure 6, and the calculation formula of the metric is shown in Table 1. In brief, this evaluation focuses on three teeth in the mandibular region (first premolar, second premolar, and first molar). We plotted the two points where each tooth contacts the alveolar bone (mesial and distal sides) and defined the midpoint between them as A. The point where a line drawn parallel to the tooth axis from point A intersects the inferior border of the mandible was designated as point B. In the central incisors, the lines were drawn parallel to the midline from the midpoints plotted on the distal surfaces of the left and right teeth, and designated as MA and MB, respectively. Measurements were taken on both the left and right sides, and the average value was used.

The changes in alveolar bone thickness are shown in Table 2. Compared to the anterior teeth, alveolar bone thickness in the molar region was more than 60% range for the first premolars and first molars at 8 years and 5 months of age; however, it was 59.6% in the second premolar region, where bone resorption was marked. The values increased with age across all regions, reaching approximately 90% by the age of 16 years and 4 months.

## 3. Discussion

Severe congenital neutropenia is a rare disorder that can lead to severe periodontitis, and dental professionals need to understand the importance of oral care in these patients. Previous reports have focused on gingival redness and alveolar bone resorption, with few studies reporting on long-term changes in the alveolar bone. We provided long-term oral management for a child with severe congenital neutropenia and confirmed improvement in the alveolar bone along with her systemic condition. The summary of the clinical timeline of the patient is shown in Table 3.

At the first visit at the age of 8 years and 5 months, only three primary second molars remained among the 20 primary teeth. Additionally, the remaining primary teeth had exposed roots, and panoramic radiographs revealed radiolucencies around the teeth and abnormal root resorption. Although there was no evidence of dental caries, there was redness and swelling of the gingiva in the incisor region, and bone resorption in both the maxilla and mandible. Bacterial plaque that forms on root surfaces exposed by periodontal disease can cause pathological changes in the pulp via lateral canals or accessory root canals, a condition known as retrograde pulpitis [12]. In our case, alveolar bone resorption associated with severe neutropenia has exposed the roots of the primary teeth. The remaining primary teeth showed no dental caries; therefore, alveolar bone resorption might have resulted from retrograde pulpitis, leading to subsequent apical periodontitis. The onset of root resorption in both the primary first and second molars is generally considered to occur around the age of 8 years [13]. However, by this age, our patient had already lost four primary first molars and one primary second molar, suggesting that these teeth may also have developed apical periodontitis, leading to tooth loss.

Although we continued follow-up of the patient, a hematopoietic stem cell transplant was scheduled due to her poor systemic condition. Patients undergoing hematopoietic stem cell transplantation are at increased risk of developing severe infection due to pre-transplant chemotherapy and post-transplant immunosuppression. Therefore, it is necessary to eliminate sources of infection before transplantation [14]. In the present case, the two remaining primary teeth were extracted prior to the transplant, and the wound healed well. The early loss of primary molars can lead to the migration of adjacent teeth, resulting in significant space loss, crowding, and potential impaction or displacement of permanent teeth; therefore, it is essential to maintain adequate space using space maintainers [15]. However, due to the poor systemic condition of our patient and the risk of causing stomatitis or mucositis by placing a space maintainer in the oral cavity, we chose observation after explaining the situation to the patient’s parents.

With continued oral care, no serious oral symptoms occurred during the hospitalization period, either before or after the transplant. At the age of 10 years, there was no redness or swelling of the gingiva, and panoramic radiography showed improvement of the alveolar bone around the erupting first premolar. Additionally, time-dependent radiographic examinations revealed similar findings around the second premolar. In addition, we measured alveolar bone thickness using panoramic images taken over time. Compared with the anterior region, alveolar bone thickness in the posterior region was approximately 60–70% at the initial visit; however, it gradually increased over time, eventually improving to about 90%. Alveolar bone growth and tooth eruption are interdependent, and the alveolar process forms during tooth development [16]. Tooth eruption or improvements in overall health may influence increases in alveolar bone volume.

Patients who receive hematopoietic stem cell transplantation at a young age have a higher risk of dental anomalies such as tooth loss, microdontia and short-rooted teeth [17,18]. Because the tooth crown and root formation progress over time, the effect on different types of teeth depends on the time of commencement of chemotherapy [18]. In the present case, the crowns of all permanent teeth had already been completed at the start of treatment. Although there was a risk of the permanent teeth developing short roots, no obvious dental abnormalities were observed. In the present case, the absence of dental abnormalities might have allowed all permanent teeth to erupt naturally.

At the age of 10 years, the patient had occlusal contact only in the incisor region. This dental alignment may have been caused by the loss of a primary molar that had been in occlusal contact and the resorption of the alveolar bone in that area. Although occlusal contact improved over time, there was still no occlusal contact between the second premolars at the age of 16. Fernandes et al. (2016) described the oral management of a patient with severe congenital neutropenia who required orthodontic treatment for maxillary atresia and anterior open bite [19]. If bone loss is significant for the patient with severe congenital neutropenia before treatment, contact with the opposing tooth may not be achieved. It is important for pediatric dentists to be aware of this and to collaborate with orthodontists as needed.

Yang et al. (2026) reported that oral management of a 12-year-old patient with acute lymphoblastic leukemia and periodontal conditions, such as probing depths and alveolar bone resorption, improved following leukemia treatment and basic periodontal therapy [9]. It suggests that reducing inflammatory burden after systemic stabilization or improving oral hygiene plays a crucial role in periodontal conditions. In addition, in the present case, the thickness of the alveolar bone was evaluated objectively rather than visually, and showed remarkable improvement as the patient’s overall condition stabilized. The degree of improvement may be influenced by various factors, such as overall health condition or age, and further comparisons focusing on each of these factors are necessary.

This report has some limitations. First, the information obtained at the first visit was insufficient because the patient’s systemic condition was very poor. For example, the intraoral photographs were limited to partial shots to minimize patient discomfort. Second, although alveolar bone thickness in the molar region improved over time, the values may depend on the imaging position. The panoramic images in this case were taken in accordance with the equipment’s imaging standards; however, they were not fully standardized. In addition, this study was a single-patient design that lacked periodontal measurements and hematologic correlation, which prevented us from demonstrating true alveolar bone regeneration. Further research is needed to quantify alveolar bone recovery in patients with neutropenia.

## Figures and Tables

**Table 1:** Evaluation metric for change in alveolar bone thickness.

Ratio	Formula (%)
First premolar	(4A to 4B)/(MA to MB) × 100
Second premolar	(5A to 5B)/(MA to MB) × 100
First molar	(6A to 6B)/(MA to MB) × 100

**Table 2:** Change in alveolar bone thickness.

	8 y 5 m	10 y	10 y 10 m	12 y 8 m	14 y 8 m	16 y 4 m
First premolar	69.9	72.9	80.6	84.0	91.4	90.1
Second premolar	59.6	62.9	66.1	77.8	88.6	88.0
First molar	62.5	67.9	73.4	77.8	85.0	88.0

**Table 3:** Chronological clinical timeline.

Age	Event	Oral Findings	Dental Intervention
8 y 5 m	Referred from Pediatrics	Redness and swelling of the gums; loosening of remaining primary teeth	Oral care during hospitalization
8 y 11 m	Due to an improvement in the overall condition, she was transferred to another hospital	The condition of the gums has improved	-
9 y 1 m	Readmission to the pediatric unit and tracheostomy for upper airway obstruction	-	Primary tooth extraction
9 y 2 m	Bone marrow transplantation	-	Oral care during hospitalization
9 y 10 m	Discharge	-	-
10 y	First visit after discharge	There were no dental caries or gingivitis; however, occlusal contact was limited to the anterior teeth	Consultation with an orthodontist at a nearby university hospital
10 y 10 m– 16 y 4 m	Regular checkups	Occlusal contact improved and alveolar bone thickness increased in the molar region	Scaling and professional mechanical tooth cleaning

## Data Availability

Data are contained within the article.
